# A phase IIb single‐center study to assess the efficacy of apremilast for the treatment of nummular eczema

**DOI:** 10.1111/ddg.15786

**Published:** 2025-06-23

**Authors:** Alexander Böhner, Peter Seiringer, Viktoria Kleeberger, Danielle Rogner, Christian Oesterlin, Tilo Biedermann, Kilian Eyerich, Felix Lauffer

**Affiliations:** ^1^ Department of Dermatology and Allergy Technical University of Munich Munich Germany; ^2^ Dermazentrum Freiburg Germany; ^3^ Department of Dermatology and Venereology Medical Center University of Freiburg Freiburg Germany; ^4^ Division of Dermatology and Venereology Department of Medicine Solna and Center for molecular medicine Karolinska Institutet Stockholm Sweden; ^5^ Department of Dermatology and Allergy LMU Hospital Munich Germany

**Keywords:** apremilast, Apremium, clinical trial, Nummular eczema, nummular dermatitis, phosphodiesterase‐4 inhibitor

## Abstract

**Background:**

The pathogenesis of nummular eczema (NE) remains unclear, and no targeted therapy has been approved. Apremilast is a small molecule inhibitor targeting phosphodiesterase‐4.

**Patients and Methods:**

A phase IIb randomized, double‐blind, placebo‐controlled study evaluating the effects of apremilast or placebo in patients with NE. Patients received apremilast (30 mg BID) or placebo until week 16 followed by an open label phase in which all patients were treated with apremilast until week 32. The primary endpoint was the number of patients achieving an improvement in *Physician's Global Assessment* (PGA) by two or more points or an absolute PGA of 0 or 1 at week 16. Secondary endpoints included changes in skin physiology, life quality, or dermato‐pathology.

**Results:**

33 patients were enrolled, of whom 31 were randomized to apremilast (n  =  15) or placebo (n  =  16). 1/15 (6.7%) patients in the apremilast group and 4/16 (25.0%) in the placebo group reached the primary endpoint (p  =  0.369). There was no difference between placebo and apremilast with regard to all secondary endpoints at week 16 and week 32. The safety profile was in accordance with the known safety profile of apremilast.

**Conclusion:**

Phosphodiesterase‐4 inhibition by apremilast showed no beneficial effects for the treatment of NE.

## INTRODUCTION

Nummular eczema (NE) is a chronic inflammatory skin disease (ISD) that is characterized by pruritic, discoid‐shaped eczematous lesions involving most frequently the upper and lower extremities.^1^, ^2^ It was first described by Devergie in 1854.^3^ Synonymous are discoid eczema, microbial eczema, and nummular dermatitis. Nummular eczema often takes a chronic and recursive course, is typically hard to treat, very itchy and severely affects quality of life.^4^ Diagnosis is made clinically in correlation with histological findings. While the etiological cause of NE is unknown, it is characterized by a combination of impaired epidermal barrier, skin inflammation, and microbial colonization.^4^, ^5^ There is an overlap with atopic dermatitis (AD), contact dermatitis, and occasionally psoriasis.^4^

Nummular eczema occurs throughout all life periods. Literature suggests a gender ratio slightly favoring women. Little is known about NE prevalence. One study reported a prevalence of up to 9% among alcoholic patients in Brazil and a point prevalence of approximately 2% in the general population.^4^ Nummular eczema is more frequent in patients with atopic disease (AD, allergic asthma, or rhinoconjunctivitis). Further trigger factors include chronic contact dermatitis, microbial skin colonization, treatment with systemic retinoids or alcohol abuse.^1^, ^2^, ^4^, ^5^, ^6^ Generalized NE has been reported in association interferon/ribavirin therapy for hepatitis C.^7^ Therapeutic options for NE are rare and include the use of emollients, topical and systemic corticosteroids, topical calcineurin inhibitors, as well as phototherapy.

Little is known about the pathophysiology of NE. Due to the association with atopic diseases, increased serum IgE levels, itch, and microbial colonization, many authors regard NE as a variant of AD. However, Noda et al. reported that the Asian variant of AD often shows clinical features similar to NE, such as sharp demarcation and nummular shaped plaque morphology. Interestingly, Asian AD clusters between psoriasis and classical AD on the level of gene regulations. Furthermore, Asian AD showed a greater acanthosis and higher turn‐over rate of keratinocyte measured by ki67 staining than classical AD. Whereas an upregulation of T helper cell (Th) 2 related genes was detected in both cases, only in skin biopsies of Asian AD was there a significant increase of IL17A, IL19, and S100A12 expression.^8^ Given the clinical similarity of NE and Asian AD it is likely that NE also relies on a mixed Th2/Th17 immune regulation. This concept is supported by the fact that NE responds well to therapies, which are effective for both psoriasis and AD, such as UV therapy and methotrexate.^9^ However, there have been neither clinical investigations of larger NE patient cohorts nor controlled clinical trials testing new compounds in NE so far. To gain more insight into the underlying immune processes of NE, data of randomized controlled trials are indispensable.

Apremilast is a small molecule that selectively inhibits the activity of phosphodiesterase 4 (PDE4), the major enzyme class responsible for the degradation of cyclic adenosine monophosphate (cAMP), an intracellular second messenger. PDE4 is expressed in inflammatory and epithelial cells, such as keratinocytes.^10^ Inhibitors of PDE4 cause accumulation of intracellular cAMP levels, which suppresses production of proinflammatory cytokines and increases production of anti‐inflammatory mediators.^11^ In contrast to other therapeutic drugs targeting directly the cytokine, PDE4‐inhibitors act at an earlier point in the inflammatory cascade.

Apremilast is approved for the treatment of plaque psoriasis, psoriatic arthritis and oral ulcers in Behçet's disease. Furthermore, there is evidence that apremilast is also effective for the treatment of AD. However, clinical trials were stopped as higher incidence of cellulitis occurred in patients receiving 40 mg apremilast twice daily (BID).^12^ Of note, immune cells in AD express high levels of PDE‐4 and murine models of AD showed good response to apremilast treatment.^13^, ^14^, ^15^ As NE shows overlapping features of both, psoriasis and AD, it was therefore hypothesized that apremilast might be effective in reducing clinical signs and symptoms of NE.

## PATIENTS AND METHODS

### Study design

**FIGURE 1:**
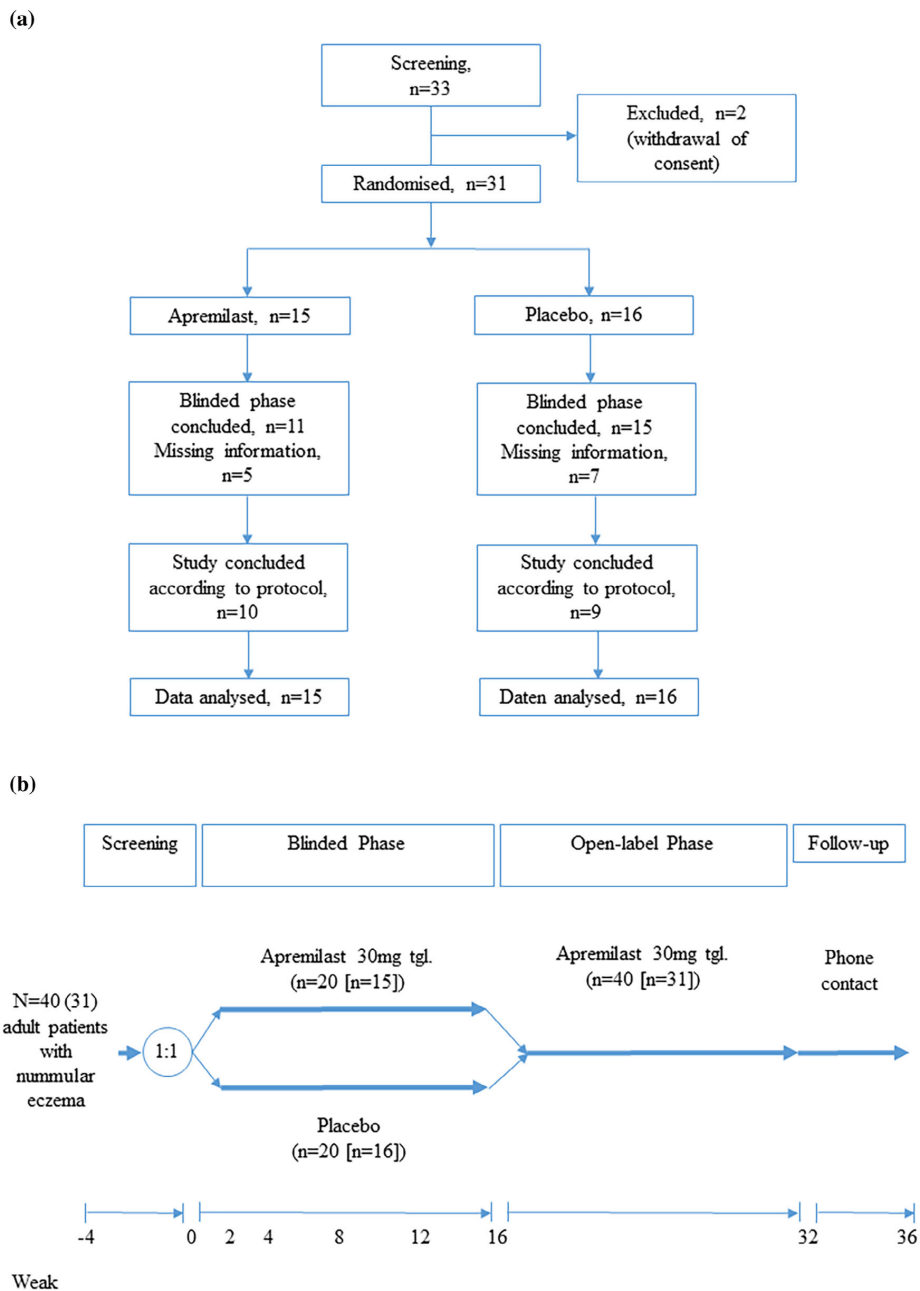
(a) Consort flow diagram of included patients; (b) study design.

We performed a single‐center, prospective, randomized, double‐blind, placebo‐controlled phase IIb study evaluating the efficacy and safety of apremilast in NE (Figure 1). Up to 40 patients were initially planned to be enrolled after written informed consent. Patients received either apremilast 30 mg BID or matching placebo in the blinded phase between week 0–16. After that, all patients were treated with apremilast 30 mg BID till week 32. During screening and treatment phase the average amount of topical corticosteroids (TCS) (class 2) was assessed. The study was approved by the responsible ethics committee and general authorities (Bundesinstitut für Arzneimittel und Medizinprodukte) and registered on clinicaltrials.gov prior to initiation (NCT03160248).

### Primary endpoint

The primary endpoint was the number of patients achieving an improvement in 5‐point *Physician's Global Assessment* (PGA) by two or more points at week 16 as compared to week 0 or achieving an absolute PGA of 0 or 1 at Week 16.

### Secondary endpoints

Secondary endpoints were assessed at week 16 and 32. These included a 50% reduction in the *Eczema Area And Severity Index* (EASI), change in *Transepidermal Water Loss* (TEWL) measured by a Tewameter^®^ TM Hex, reduction of acanthosis or the number inflammatory cells in skin biopsies as assessed by a certified dermato‐pathologist, change in the amount of TCS, change in PGA, change in the *Dermatology Life Quality Index* (DLQI), change in pruritus *Visual Analogue Scale* (VAS), change in the global satisfaction subscale of the *Treatment Satisfaction Questionnaire for Medication* (TSQM) and safety.

### Inclusion and exclusion criteria

Key inclusion criteria were clinically and histologically confirmed moderate or severe NE (PGA 3–5), which were insufficiently controlled by TCS for at least 8 weeks. The minimum disease duration was three months. Histological diagnosis of eczema was made at the discretion of the dermato‐pathologist and included the hallmarks spongiosis, eosinophils, serum crusts, and (irregular) acanthosis as well as absence of hallmarks of a differential diagnosis (e.g., Munro microabscesses). Key exclusion criteria were concomitant severe diseases, such as malignancies, immunological disorders, neurological disorders, depression or suicidal ideation, cardiovascular disease, severe renal or hepatic insufficiencies.

### Sample size calculation

Assuming an improvement of 2 points (standard deviation 1.7) in the apremilast group versus 0 points in the placebo group, a sample size of 17 patients per arm was estimated to provide 85% power to detect a difference in median PGA scores using a two‐sided Wilcoxon test at a 5% significance level. Accounting for an estimated dropout rate of 15%, a total of 40 patients (20 per group) was planned for enrollment.

### Statistical analysis plan

All patients who received at least one dose of study medication were included in the final analysis. For the primary endpoint the absolute change in PGA score at week 16 was compared between apremilast and placebo group using the two‐sided Wilcoxon test with a significance level of 5%. Missing data was imputed using the highest documented change in PGA in the placebo group and 0 in the apremilast group.

## RESULTS

### Patient's demographics and baseline characteristics

According to the study protocol, enrollment of 40 patients was planned. However, due to delays in recruitment, the lead investigators decided to discontinue further enrollment after 33 patients had been included. Two patients withdrew consent before randomization. Thirty‐one patients entered the treatment period (15 in the apremilast arm, 15 in the placebo arm). Nineteen patients participated till the end of the study, while 12 patients discontinued prematurely due to adverse events (n  =  5) or withdrawal of consent (n  =  7; 5 in the apremilast arm, 7 in the placebo arm). In the apremilast and placebo + apremilast groups, 11 of 15 patients (73.3%) and 15 of 16 patients (93.8%), respectively, completed the blinded phase, while 10 of 15 (66.7%) and 9 of 16 (56.3%) reached the end of the open‐label phase. There were no significant differences in mean PGA scores or baseline characteristics between apremilast and placebo arm (Table 1).

**TABLE 1:** Demographics and baseline characteristics.

		Treatment group			
		*Apremilast (n = 15)*		*Placebo + Apremilast (n = 16)*	
Sex (n, %)	Female	5	(33.3%)	2	(12.5%)
	Male	10	(66.7%)	14	(87.5%)
Age group (n, %)	Adults (18–64 years)	11	(73.3%)	11	(68.8%)
	Elderly (65–84 years)	4	(26.7%)	5	(31.5%)
Age (years)	Mean	54.5		54.6	
	Std	17.0		15.5	
	Min	27.0		23.0	
	Median	62.0		55.5	
	Max	81.0		78.0	
Ethnicity (n, %)	Asian	1	(6.7%)	1	(6.3%)
	Caucasian	13	(86.7%)	15	(93.8%)
	Other	1	(6.7%)	0	
BMI	Mean	28.7		28.6	
	Std.	4.8		6.0	
PGA	3	12	(80.0%)	15	(20.0%)
	4	3	(20.0%)	1	(6.3%)

### Apremilast is not an efficacious treatment for nummular eczema

There was no statistical difference between the apremilast arm and the placebo arm with regard to the primary endpoint (2 points PGA improvement till week 16). Due to missing values, PGA at week 16 was imputed for four patients as described in the Methods section. An improvement in PGA was observed in 1/15 (6.7%) patients in the apremilast group and 4/16 (25.0%) patients in the placebo group (p  =  0.369) (Table 2). When analyzing completed cases only without imputation, there was PGA improvement in 1/11 (9.1%) in the apremilast and 2/14 (14.3%) in the placebo + apremilast group (p  =  0.812) (online supplementary Table S1). Thus, there was no difference in PGA improvement between patients treated with apremilast and patients receiving placebo. This study included a number of secondary endpoints, such as EASI50 (Table 2), the improvement of skin inflammation in skin biopsies at week 0 and 16, changes in daily use of topical corticosteroids, life quality, pruritus and transepidermal water loss as well as the overall treatment satisfaction with apremilast measured by the *Treatment Satisfaction Questionnaire for Medication* (TSQM). None of these secondary endpoints showed a difference between apremilast and placebo at week 16 or week 32 (online supplementary Tables S2–S8). In summary, there is no evidence for an efficacy of apremilast in NE.

**TABLE 2:** Primary endpoint: *Physician's Global Assessment* (PGA) as well as 50%. improvement of the *Eczema Area and Severity Index* (EASI50) score at week 16 and 32.

PGA improvement from baseline						*p value*
		*Apremilast*		*Placebo + Apremilast*		
		(n = 15)		(n = 16)		
Week 16	No improvement	14	(93.3%)	12	(75.0%)	0.369
	Improvement	1	(6.7%)	4	(25.0%)	

EASI 50 improvement from Baseline to						*p value*
		*Apremilast*		*Placebo + Apremilast*		
		(n = 11)		(n = 14)		
Week 16	No improvement	6	(54.5%)	8	(57.1%)	1.000
	Improvement	5	(45.5%)	6	(42.9%)	
		(n = 11)		(n = 11)		
Week 32	No improvement	6	(54.5%)	5	(45.5%)	0.670
	Improvement	5	(45.5%)	6	(54.5%)	

### Apremilast was well tolerated, and no new safety signal occurred

A total of 59 adverse events (AE) were reported. 37.3% AEs were deemed related to treatment administered. During blinded phase there were 22 AEs in the apremilast arm and 21 AEs in the placebo arm. During the open‐label‐phase additional 16 AEs were reported. One serious AE occurred in the apremilast arm during the blinded phase, when one patient was hospitalized due to worsening of NE (Table 3). The majority of adverse events (AEs) were rated as mild (61%) or moderate (35.6%), while only two AEs (3.4%) were rated as severe. No suspected unexpected serious adverse events, life‐threatening or fatal adverse events occurred. Patients treated with apremilast had higher rates of diarrhea, nausea, and viral upper respiratory infections than patients receiving placebo. A detailed list of all AEs can be found in online supplementary Tables S9 and S10. The overall safety profile was in line with the previously described safety profile of apremilast.

**TABLE 3:** Adverse events (AE) and serious adverse events (SAE).

	Blinded phase						Open label phase		
	Exposed to Apremilast (n = 15)			Exposed to Placebo (n = 16)			Exposed to Apremilast (n = 25)		
		Subjects affected			Subjects affected			Subjects affected	
	*Events*	*n*	*(%)*	*Events*	*n*	*(%)*	*Events*	*n*	*(%)*
All AEs	22	10	(67)	21	10	(63)	16	9	(36)
SAE	1	1	(7)	0	0		0	0	
Non‐SAE AEs	21	10	(67)	21	10	(63)	16	9	(36)

## DISCUSSION

Inflammatory skin diseases seriously constrain patient's quality of life.^16^, ^17^ While there have been numerous approvals of new compounds for the treatment of the two most common ISD, psoriasis and AD, the vast majority of ISD remain a therapeutic challenge.^18^ Nummular eczema is a prominent example of an ISD but has been poorly investigated despite its high prevalence in children and adults. While there is no doubt that NE is a relevant ISD with a high unmet need for new treatment options, investigating NE is complicated by an inconsistent definition, use of multiple synonyms, and limited data available in the literature.

This is the first randomized controlled clinical trial conducted in patients with NE. Patients received 30 mg apremilast BID or placebo for a 16‐week blinded phase, followed by a 16‐week open‐label phase. As no clinical score for NE exists, significant improvement in 5‐point PGA was defined as the primary endpoint. Several secondary endpoints were included to assess the presence of any additional, or more subtle, differences between placebo and apremilast treatment at different timepoints. Apremilast demonstrated no efficacy in NE, neither in terms of PGA improvement nor in relation to changes in disease‐related symptoms, quality of life, or skin histology.

Our study has limitations. First the recruitment goal of 40 patients was not met. However, it is not expected that the clear results obtained would change significantly with a higher number of patients. Second, there was a high drop‐out rate, which is most likely based on the low efficacy. Lastly, this is a single center study in Germany not representing the spectrum of different ethnicities. Nevertheless, results of this study are highly relevant as they greatly contribute to the ongoing debate of whether NE is either a variant of AD with a predominant Th2 immune response, a mixed Th2/Th17 phenotype, or a distinct entity with an immunopathogenesis still to be discovered.

Nummular eczema shares clinical, histological, and molecular hallmarks of both type 2 (Th2) and type 3 (Th17) immunity.^19^ Clinically, NE shares the sharp demarcation and the affected body sites with psoriasis, while single lesions often show papules and serum crusts. Histological changes of NE consist of hallmarks of both eczema (spongiosis, eosinophils) and psoriasis (acanthosis, neutrophils). Pruritus and microbial colonization are indicative of an eczema variant.^4^, ^5^, ^20^, ^21^, ^22^ From an immunological point of view, both type 2‐associated genes such as *IL‐13*, *CCL17*, *CCL18*, and *CCL26*, as well as type 3 immune genes such as *IL‐19*, *CXCL8*, and *CXCL5*, are upregulated in NE lesional skin.^19^ However, whether Th2 and Th17 immunity are co‐dominant or whether there is one immune signature driving NE pathogenesis remains open. Here, only data from randomized controlled clinical trials can definitely prove or disprove the impact of different immune mechanisms.

In our study we tested the efficacy and safety of apremilast, a small molecule inhibiting PDE4 and thereby dampening Th17 immunity as well as NF‐kB driven inflammation. Given the lack of efficacy in NE and the fact that apremilast is approved for the treatment of plaque psoriasis, a prototype of Th17 mediated skin diseases, we can conclude that Th17 immunity does not play the dominant role in the immunopathogenesis of NE. This observation is in line with clinical trials testing apremilast in AD. Here, only the highest dosage of 40 mg apremilast BID showed clinical improvements. However, this arm had to be discontinued due to safety concerns.^12^ Based on our data, new therapeutic options for NE appear feasible. Inhibition of Th2 cytokines by dupilumab, an IL‐4/IL‐13 receptor antagonist, or the anti‐IL‐13 antibodies tralokinumab or lebrikizumab, seems promising. Likewise, the use of Janus kinase inhibitors approved for the treatment of atopic dermatitis – such as baricitinib, upadacitinib, and abrocitinib – might be beneficial. Of note, an investigator‐initiated clinical trial testing the efficacy of dupilumab for NE is currently running (clinicaltrials.gov identifier: NCT04600362).

In summary we demonstrated that apremilast is not effective in the treatment of NE, thereby proving that potential activation of Th17 immunity is not causative for NE development or maintenance. Other treatment regimens inhibiting Th2 immunity appear more promising and future studies are needed to define better treatment strategies for NE.

## CONFLICT OF INTEREST STATEMENT

The authors declare no conflict of interest.

## Supporting information



Supplementary information

## References

[1] Bonamonte D , Foti C , Vestita M , et al. Nummular eczema and contact allergy: a retrospective study. Dermatitis. 2012;23:153‐157. 22828253 10.1097/DER.0b013e318260d5a0

[2] Halberg M . Nummular eczema. J Emerg Med. 2012;43:e327‐e328. 21719233 10.1016/j.jemermed.2011.05.031

[3] Devergie M‐G‐A . Traité pratique des maladies de la peau, 1854.

[4] Jiamton S , Tangjaturonrusamee C , Kulthanan K . Clinical features and aggravating factors in nummular eczema in Thais. Asian Pac J Allergy Immunol. 2013;31:36‐42. 23517392

[5] Kim WJ , Ko HC , Kim MB , et al. Features of *Staphylococcus aureus* colonization in patients with nummular eczema. Br J Dermatol. 2013;168:658‐660. 22630164 10.1111/j.1365-2133.2012.11072.x

[6] Silverberg JI , Hou A , Warshaw EM , et al. Prevalence and trend of allergen sensitization in patients with nummular (discoid) eczema referred for patch testing: North American Contact Dermatitis Group data, 2001–2016. Contact Dermatitis. 2021;85:46‐57. 33634511 10.1111/cod.13824

[7] Shen Y , Pielop J , Hsu S . Generalized nummular eczema secondary to peginterferon Alfa‐2b and ribavirin combination therapy for hepatitis C infection. Arch Dermatol. 2005;141: 102‐103. 10.1001/archderm.141.1.102-b 15655158

[8] Noda S , Suarez‐Farinas M , Ungar B , et al. The Asian atopic dermatitis phenotype combines features of atopic dermatitis and psoriasis with increased TH17 polarization. J Allergy Clin Immunol. 2015;136:1254‐1264. 26428954 10.1016/j.jaci.2015.08.015

[9] Roberts H , Orchard D . Methotrexate is a safe and effective treatment for paediatric discoid (nummular) eczema: a case series of 25 children. Australas J Dermatol. 2010;51:128‐130. 20546220 10.1111/j.1440-0960.2010.00634.x

[10] Moustafa F , Feldman SR . A review of phosphodiesterase‐inhibition and the potential role for phosphodiesterase 4‐inhibitors in clinical dermatology. Dermatol Online J. 2014;20: 22608. 24852768

[11] Schett G . Apremilast in psoriatic arthritis. Clin Exp Rheumatol. 2015;33:S98‐S100. 26472278

[12] Simpson EL , Imafuku S , Poulin Y , et al. A Phase 2 Randomized Trial of Apremilast in Patients with Atopic Dermatitis. J Invest Dermatol. 2019;139:1063‐1072. 30528828 10.1016/j.jid.2018.10.043

[13] Chan SC , Reifsnyder D , Beavo JA , Hanifin JM . Immunochemical characterization of the distinct monocyte cyclic AMP‐phosphodiesterase from patients with atopic dermatitis. J allergy Clin Immunol. 1993;91:1179‐1188. 8389777 10.1016/0091-6749(93)90321-6

[14] Baumer W , Hoppmann J , Rundfeldt C , Kietzmann M . Highly selective phosphodiesterase 4 inhibitors for the treatment of allergic skin diseases and psoriasis. Inflamm Allergy Drug Targets. 2007;6:17‐26. 17352685 10.2174/187152807780077318

[15] Schafer PH , Adams M , Horan G , et al. Apremilast Normalizes Gene Expression of Inflammatory Mediators in Human Keratinocytes and Reduces Antigen‐Induced Atopic Dermatitis in Mice. Drugs R D. 2019;19:329‐338. 31598889 10.1007/s40268-019-00284-1 PMC6890576

[16] Misery L , Seneschal J , Reguiai Z , et al. Patient Burden is Associated with Alterations in Quality of Life in Adult Patients with Atopic Dermatitis: Results from the ECLA Study. Acta Derm Venereol. 2018;98(7):713‐714. 29648674 10.2340/00015555-2940

[17] Langenbruch A , Radtke MA , Jacobi A , et al. Quality of psoriasis care in Germany: results of the national health care study “PsoHealth3”. Arch Dermatol Res. 2016;308:401‐408. 27206971 10.1007/s00403-016-1651-x

[18] Eyerich K , Eyerich S . Immune response patterns in non‐communicable inflammatory skin diseases. J Eur Acad Dermatol Venereol. 2018;32:692‐703. 29114938 10.1111/jdv.14673 PMC5947562

[19] Bohner A , Jargosch M , Muller NS , et al. The neglected twin: Nummular eczema is a variant of atopic dermatitis with codominant T(H)2/T(H)17 immune response. J Allergy Clin Immunol. 2023;152:408‐419 37119871 10.1016/j.jaci.2023.04.009

[20] Rendon A , Schakel K . Psoriasis Pathogenesis and Treatment. Int J Mol Sci. 2019;20. 10.3390/ijms20061475 PMC6471628 30909615

[21] Kong HH , Oh J , Deming C , Conlan S , et al. Temporal shifts in the skin microbiome associated with disease flares and treatment in children with atopic dermatitis. Genome Res. 2012;22:850‐859. 22310478 10.1101/gr.131029.111 PMC3337431

[22] Weidinger S , Novak N . Atopic dermatitis. Lancet. 2016;387:1109‐1122. 26377142 10.1016/S0140-6736(15)00149-X

