# Supplementary material for: A phase IIb single‐center study to assess the efficacy of apremilast for the treatment of nummular eczema
Source: J Dtsch Dermatol Ges. 2025 Jun 23;23(8):959–65. doi: 10.1111/ddg.15786 (PMC12338434; doi:10.1111/ddg.15786)
Supplement: Supplementary file 1 — Supplementary information [file DDG-23-959-s001.docx]

**[[Supplementary tables]]**

Supplementary TABLE S1 Improvement of *Physician’s Global Assessment* (PGA), completed cases till week 16 only.

Table  S1

| **PGA improvement**  **from Baseline** | | **Treatment group** | | | |  |
| --- | --- | --- | --- | --- | --- | --- |
|  |  | ***Apremilast*** | | ***Placebo + Apremilast*** | | **p value** |
|  |  | (n = 11) | | (n = 14) | |  |
| Week 16  (Complete case) | No improvement | 10 | (90.9%) | 12 | (85.7%) | 0.812 |
|  | Improvement | 1 | (9.1%) | 2 | (14.3%) |  |

Supplementary TABLE S2 Change in mean *Transepidermal Water Loss* (TEWL).

Table  S2

| **Change in mean TEWL between baseline and …** | |  | | **p value** |
| --- | --- | --- | --- | --- |
|  |  | **Apremilast** | **Placebo + Apremilast** |  |
| Week 16 | *n* | 9 | 9 | 0.185 |
|  | *Mean* | –26.04 | –12.24 |  |
|  | *Std* | 10.29 | 21.47 |  |
| Week 32 | *n* | 8 | 6 | 0.847 |
|  | *Mean* | –19.08 | –16.85 |  |
|  | *Std* | 19.46 | 29.73 |  |

Supplementary TABLE S3 Histological improvement.

Table  S3

| **Histological improvement** | |  | | **p value**  **(Fisher Exact Test)** |
| --- | --- | --- | --- | --- |
|  |  | **Apremilast** | **Placebo + Apremilast** |  |
| Overall histological improvement  at Week 16 | *No improvement* | 7 (63.6%) | 9 (64.3%) | 1.000 |
|  | *Improvement* | 4 (36.4%) | 5 (35.7%) |  |
| Epidermal thickness at Baseline | *n* | 11 | 14 |  |
|  | *Mean* | 318.73 | 291.29 |  |
|  | *Std* | 88.84 | 114.44 |  |
| Epidermal thickness at Week 16 | *n* | 11 | 14 |  |
|  | *Mean* | 230.32 | 200.57 |  |
|  | *Std* | 59.29 | 127.07 |  |
| Immune cell count at Baseline | *n* | 11 | 14 |  |
|  | *Mean* | 192.77 | 193.11 |  |
|  | *Std* | 59.08 | 69.08 |  |
| Immune cell count at Week 16 | *n* | 11 | 14 |  |
|  | *Mean* | 115.55 | 112.00 |  |
|  | *Std* | 82.74 | 89.38 |  |

Supplementary TABLE S4 Mean daily use of topical steroids (g).

Table  S4

| **Mean daily steroid use during …** | |  | | **p value** |
| --- | --- | --- | --- | --- |
|  |  | **Apremilast** | **Placebo + Apremilast** |  |
| Screening | *n* | 13 | 13 | 0.538 |
|  | *Mean* | 4.23 | 2.63 |  |
|  | *Std* | 3.79 | 1.44 |  |
| Phase I (until week 16) | *n* | 13 | 15 | 0.854 |
|  | *Mean* | 3.18 | 2.84 |  |
|  | *Std* | 3.02 | 1.85 |  |
| Phase II (week 16–week 32) | *n* | 11 | 10 | 0.597 |
|  | *Mean* | 1.31 | 1.61 |  |
|  | *Std* | 1.10 | 1.10 |  |

Supplementary TABLE S5 *Dermatology Life Quality Index* (DLQI).

Table  S5

| **Change in DLQI between baseline and …** | |  | | **p value** |
| --- | --- | --- | --- | --- |
|  |  | **Apremilast** | **Placebo + Apremilast** |  |
| Week 16 | *n* | 11 | 14 | 0.678 |
|  | *Mean* | –3.82 | –2.36 |  |
|  | *Std* | 4.92 | 3.39 |  |
| Week 32 | *n* | 11 | 11 | 0.792 |
|  | *Mean* | –4.45 | –4.64 |  |
|  | *Std* | 9.46 | 5.71 |  |

Supplementary TABLE S6 Pruritus *VISUAL ANALOGUE SCALE* (VAS) at week 16 and 32.

Table  S6

| **Change in VAS between baseline and …** | |  | | **p value** |
| --- | --- | --- | --- | --- |
|  |  | **Apremilast** | **Placebo + Apremilast** |  |
| *Week 16* | *n* | 11 | 14 | 0.617 |
|  | *Mean* | –0.27 | –0.18 |  |
|  | *Std* | 2.38 | 1.69 |  |
| *Week 32* | *n* | 11 | 11 | 0.741 |
|  | *Mean* | –1.00 | –1.55 |  |
|  | *Std* | 4.04 | 2.94 |  |

Supplementary TABLE S7 *Treatment Satisfaction Questionnaire for Medication* (TSQM) score.

Table  S7

| **Change in TSQM sub-score between Baseline and …** | |  | | **p value** |
| --- | --- | --- | --- | --- |
|  |  | **Apremilast** | **Placebo + Apremilast** |  |
| Effectiveness  Week 16 | *n* | 9 | 10 | 0.152 |
|  | *Mean* | –3.71 | –12.24 |  |
|  | *Std* | 20.22 | 30.75 |  |
| Effectiveness  Week 32 | *n* | 9 | 8 | 0.194 |
|  | *Mean* | –0.01 | 19.84 |  |
|  | *Std* | 32.62 | 24.44 |  |
| Side effects  Week 16 | *n* | 9 | 10 | 0.428 |
|  | *Mean* | –6.94 | 2.50 |  |
|  | *Std* | 52.70 | 29.93 |  |
| Side effects  Week 32 | *n* | 9 | 8 | 0.285 |
|  | *Mean* | –13.88 | –34.11 |  |
|  | *Std* | 52.73 | 37.75 |  |
| Convenience  Week 16 | *n* | 9 | 10 | 0.775 |
|  | *Mean* | 15.42 | 10.04 |  |
|  | *Std* | 32.11 | 29.32 |  |
| Convenience  Week 32 | *n* | 9 | 8 | 0.661 |
|  | *Mean* | 9.87 | 14.59 |  |
|  | *Std* | 48.00 | 26.76 |  |
| Global satisfaction Week 16 | *n* | 9 | 10 | 0.367 |
|  | *Mean* | –10.31 | –21.36 |  |
|  | *Std* | 25.77 | 42.59 |  |
| Global satisfaction Week 32 | *n* | 9 | 8 | 0.665 |
|  | *Mean* | –2.38 | 2.72 |  |
|  | *Std* | 37.61 | 36.63 |  |

Supplementary TABLE S8 Changes of *Physician’s Global Assessment* (PGA) between blinded and open label phase.

Table  S8

| **Change in PGA between …** | |  | |
| --- | --- | --- | --- |
|  |  | **Apremilast** | **Placebo + Apremilast** |
| p value (Wilcoxon Signed Ranks Test)  Comparing blinded and unblended period within treatment group | | 0.391 | 0.625 |
| Baseline and Week 16 | *n* | 11 | 14 |
|  | *Mean* | –0.73 | –0.57 |
|  | *Std* | 0.90 | 0.85 |
| Baseline and Week 32 | *n* | 11 | 11 |
|  | *Mean* | –0.73 | –0.82 |
|  | *Std* | 0.65 | 0.87 |
| Week 16 and Week 32 | *n* | 11 | 11 |
|  | *Mean* | 0.00 | –0.55 |
|  | *Std* | 1.18 | 0.82 |

Supplementary TABLE S9 Non-serious adverse events – blinded phase.

Table  S9

|  | **Exposed to Apremilast n = 15**  **______________** | | | **Exposed to Placebo n = 16**  **_______________** | | |
| --- | --- | --- | --- | --- | --- | --- |
| **System Organ Class Preferred Term** |  | ***Subjects affected***  ***________*** | |  | ***Subjects affected ________*** | |
|  | *Events* | *n* | *(%)* | *Events* | *n* | *(%)* |
| ***OVERALL*** | 21 | 10 | (67) | 21 | 10 | (63) |
| ***Gastrointestinal disorders*** | 9 | 7 | (47) | 2 | 2 | (13) |
| Abdominal distension | 1 | 1 | (7) | 0 | 0 | (0) |
| Abdominal pain upper | 1 | 1 | (7) | 0 | 0 | (0) |
| Diarrhea | 3 | 3 | (20) | 1 | 1 | (6) |
| Flatulence | 1 | 1 | (7) | 0 | 0 | (0) |
| Nausea | 3 | 3 | (20) | 1 | 1 | (6) |
| ***General disorders and administration site conditions*** | 2 | 1 | (7) | 1 | 1 | (6) |
| Chest pain | 0 | 0 | (0) | 1 | 1 | (6) |
| Fatigue | 2 | 1 | (7) | 0 | 0 | (0) |
| ***Infections and infestations*** | 7 | 7 | (47) | 4 | 3 | (19) |
| Hordeolum | 1 | 1 | (7) | 0 | 0 | (0) |
| Pneumonia | 0 | 0 | (0) | 1 | 1 | (6) |
| Viral upper respiratory tract infection | 6 | 6 | (40) | 3 | 3 | (19) |
| ***Investigations*** | 1 | 1 | (7) | 0 | 0 | (0) |
| Hepatic enzyme increased | 1 | 1 | (7) | 0 | 0 | (0) |
| ***Metabolism and nutrition disorders*** | 1 | 1 | (7) | 0 | 0 | (0) |
| Decreased appetite | 1 | 1 | (7) | 0 | 0 | (0) |
| ***Musculoskeletal and connective tissue disorders*** | 0 | 0 | (0) | 1 | 1 | (6) |
| Musculoskeletal pain | 0 | 0 | (0) | 1 | 1 | (6) |
| ***Neoplasms benign, malignant and unspecified (including cysts and polyps)*** | 0 | 0 | (0) | 1 | 1 | (6) |
| Keratoacanthoma | 0 | 0 | (0) | 1 | 1 | (6) |
| ***Nervous system disorders*** | 1 | 1 | (7) | 3 | 2 | (13) |
| Dizziness | 1 | 1 | (7) | 0 | 0 | (0) |
| Headache | 0 | 0 | (0) | 1 | 1 | (6) |
| Migraine | 0 | 0 | (0) | 2 | 1 | (6) |
| ***Skin and subcutaneous tissue disorders*** | 0 | 0 | (0) | 9 | 3 | (19) |
| Eczema | 0 | 0 | (0) | 1 | 1 | (6) |
| Eczema weeping | 0 | 0 | (0) | 4 | 1 | (6) |
| Pruritus | 0 | 0 | (0) | 4 | 2 | (13) |

Supplementary TABLE S10 Non-serious adverse events – open label phase.

Table  S10

|  | **Exposed to**  **Apremilast n = 25** *_______________* | | |
| --- | --- | --- | --- |
| **System Organ Class  Preferred Term** |  | ***Subjects affected*** *________* | |
|  | *Events* | *n* | *(%)* |
| **OVERALL** | 16 | 9 | (36) |
| ***Eye disorders*** | 1 | 1 | (4) |
| Cataract | 1 | 1 | (4) |
| ***Gastrointestinal disorders*** | 6 | 4 | (16) |
| Abdominal discomfort | 1 | 1 | (4) |
| Diarrhea | 3 | 2 | (8) |
| Nausea | 1 | 1 | (4) |
| Vomiting | 1 | 1 | (4) |
| ***General disorders and administration site conditions*** | 1 | 1 | (4) |
| Oedema peripheral | 1 | 1 | (4) |
| ***Infections and infestations*** | 2 | 2 | (8) |
| Viral upper respiratory tract infection | 2 | 2 | (8) |
| ***Nervous system disorders*** | 2 | 2 | (8) |
| Headache | 2 | 2 | (8) |
| ***Skin and subcutaneous tissue disorders*** | 4 | 2 | (8) |
| Eczema | 1 | 1 | (4) |
| Photodermatoses | 1 | 1 | (4) |
| Pruritus | 2 | 2 | (8) |
